# Pancreatic ductal cells may have a negative effect on human islet transplantation

**DOI:** 10.1371/journal.pone.0220064

**Published:** 2019-07-19

**Authors:** Sandra Marín-Cañas, Elisabet Estil·les, Laura Llado, Patricia San José, Montserrat Nacher, Noèlia Téllez, Eduard Montanya

**Affiliations:** 1 Bellvitge Biomedical Research Institute (IDIBELL), L’Hospitalet de Llobregat, Barcelona, Spain; 2 CIBER of Diabetes and Associated Metabolic Diseases (CIBERDEM), Barcelona, Spain; 3 Department of Clinical Science, University of Barcelona, L’Hospitalet de Llobregat, Barcelona, Spain; 4 Liver Transplant Unit, University Hospital of Bellvitge, L'Hospitalet de Llobregat, Barcelona, Spain; 5 Endocrine Unit, University Hospital of Bellvitge, L’Hospitalet de Llobregat, Barcelona, Spain; Vrije Universiteit Brussel, BELGIUM

## Abstract

**Aim:**

To evaluate the effect of pancreatic ductal cells on experimental human islet transplantation.

**Materials and methods:**

Isolated islets were additionally purified by handpicking. Ductal cells were purified by magnetic cell sorting and then clustered into ductal pancreatospheres (DPS). Islets, DPS, and islets + DPS (100 islets + 75 DPS, or 100 islets + 200 DPS) were cultured and glucose-stimulated insulin secretion, β-cell apoptosis, and gene expression was determined. Islets and islets + DPS preparations (800 islets + 600 DPS) were transplanted to streptozotocin-treated immunodeficient mice and glycemia, graft morphometry, and gene expression were determined.

**Results:**

Insulin stimulation index was higher in islets than in islets co-cultured with DPS (5.59 ± 0.93 *vs* 4.02 ± 0.46; *p*<0.05). *IL1B* and *CXCL11* expression was higher in 100 islets + 200 DPS than in islets (*p*<0.01), and IL-1β was detected in supernatants collected from DPS and islets + DPS preparations, but not in islets. Hyperglycemia developed in 33% and 67% of mice transplanted with islets or with islets + DPS respectively. β-cell mass was 26% lower in islets + DPS than in islets grafts (p>0.05), and the ratio β-/endocrine non-β-cell mass was lower in islets + DPS grafts (islets: 2.05 ± 0.18, islets + DPS: 1.35 ± 0.15; *p*<0.01). *IL1B* and *IL1RN* expression was significantly higher in islets + DPS grafts.

**Conclusions:**

Islet preparations enriched with ductal cells have a lower insulin stimulation index *in vitro* and achieved a worse metabolic outcome after transplantation. Inflammation may mediate the deleterious effects of ductal cells on islet cells.

## Introduction

Islet transplantation is an effective and safe treatment for type 1 diabetic patients with hypoglycemia unawareness and severe hypoglycemic events [1]. The scarce availability of pancreata from human organ donors and the need for chronic immunosuppression restrict the clinical use of islet transplantation [2]. The success of islet transplantation is limited by primary non-function of islet grafts, and by the progressive loss of islet function that results in the recurrence of hyperglycemia [3,4]. The characteristics of the transplanted preparation, and in particular the cell composition, is an important factor determining the success of the procedure [5]. Different contaminating cell types may have diverging effects on transplanted islets. Acinar cells secrete proteolytic enzymes that create a dysfunctional environment for islets [6], and exocrine contamination can impair the implantation of islets, increase the inflammatory response and impair the revascularization of the graft [7–9]. In contrast, co-transplantation of mesenchymal stem cells [10] or endothelial progenitor cells [11] may improve the efficacy of islet transplantation.

Ductal cells may account for up to 40% of the cells in human islet preparations [12,13], but their role on islet transplantation outcome has not yet been established. There are evidences supporting both potentially beneficial and deleterious effects of ductal cells on transplanted islets. Ductal cells produce proinflammatory mediators, such as IL-1β, TNF-α, CD40, nitric oxide and tissue factor [14–18] that could impair β-cell function and increase β-cell loss in transplanted islets. On the other hand, ductal cells are a source of proangiogenic cytokines IL-8 and VEGF that could enhance the revascularization of the graft and improve the survival of transplanted islets [19,20]. Moreover, pancreatic ductal epithelium may harbor a pool of islet progenitors [21] that might contribute to maintain or even increase the graft β-cell mass. *In vitro* studies have described beneficial, neutral and deleterious effects of ductal cells on islet cell viability and function [14–19,22,23], and the limited number of islet transplantation studies have also yielded variable results. In rodents, exocrine contamination showed a negative effect on islet transplantation [8], but ductal cells were suggested to have a positive effect on graft function [20]. These studies were based on the metabolic evolution after transplantation, with no direct assessment of the grafts. Human ductal cells did not modify the islet graft outcome in rodents [16], although data from clinical studies have suggested a positive effect [12,24]. Overall, current evidence about the effects of ductal cells present in islet preparations is scarce and controversial. In this study we aimed to determine the effect of pancreatic ductal cells on islet function and on islet transplantation using well-characterized human islet and ductal cell preparations.

## Materials and methods

### Islet isolation

Human pancreatic islets were isolated from 21 non-diabetic adult deceased organ donors, 14 males, 58.8 ± 2.7 years (range: 32–80), BMI 26.2 ± 0.86 (Kg/m^2^), by collagenase digestion (Collagenase HA with thermolysin and clostripain, VitaCyte, Indianapolis, IN, USA) using the Ricordi method and purified on a refrigerated COBE 2991 cell processor (COBE BCT, Laekwood, CO, USA), as previously described [25]. After islet isolation, the islet-enriched and islet-depleted fractions were collected and processed separately. Islet-enriched fractions were cultured in CMRL-1066 medium (5.6 mM glucose) (Connaght Medical Research Laboratories; Mediatech, Manassas, VA, USA) supplemented with 10% ABO compatible human serum (Blood and Tissue Bank, Barcelona, Spain), 2 mM L-glutamine (Life Technologies, Grand Island, NY, USA), 10 mM nicotinamide (Sigma-Aldrich, St. Louis, MO, USA), 10 mM HEPES (Biological Industries, Kibbutz BeitHaemek, Israel), 250 ng/ml amphotericin B (Fungizone, Life Technologies), 40 μg/ml gentamicin (Laboratorios Normon, Madrid, Spain) and 20 μg/ml ciprofloxacin (Fresenius-Kabi, Barcelona, Spain) at 37°C and 5% CO_2_ for 4–6 days, and culture media was changed every two days. To further improve the islet purity of the preparations, after 4–6 days in culture islets were handpicked using a low dithizone (Sigma-Aldrich) concentration (0.023 mM) that does not impair β-cell function [26] and were used for *in vitro* or for islet transplantation experiments. Use of human islets was approved by the local Ethics Committee of Hospital Universitari Bellvitge (identification number: PR239/13), and signed consent was obtained from donor relatives.

### Ductal cell purification

The exocrine, islet-depleted, fractions left after islet isolation and purification were cultured overnight in CMRL 1066 medium supplemented with 0.5% human serum albumin (Grifols, Barcelona, Spain). These islet-depleted preparations were then dispersed into single cells with 0.16 g/l trypsin (Sigma-Aldrich), 0.1 mM EDTA (Sigma-Aldrich) and 10 ng/ml DNAse (Pulmozyme, Roche Diagnostics, Mannheim, Germany), filtered through 60 μm cell strainer (Merck Millipore, Billerica, MA, USA), and ductal cells were subsequently purified by magnetic-activated cell sorting (MACS). Dispersed single cells were labeled with mouse carbohydrate antigen 19–9 (CA19-9) antibody [27] (Clone C241:5:1:4; Leica microsystems, Wetzlar, Germany) (final dilution 1:200) and with rat anti-mouse IgG microbeads (Miltenyi Biotec, Bergisch Gladbach, Germany) (final dilution 1:5). CA19-9 labeled cells were separated using magnetic LS separation columns (Miltenyi Biotec) according to the manufacturer’s instructions. The magnetic field was removed and the CA19-9 positive ductal cell fraction was collected. One million viable cells per well were seeded in ultralow attachment six-well plates (Corning, Corning, NY, USA). Cells were cultured in serum-free Dulbecco's Modified Eagle’s Medium/Ham’s Nutrient Mixture F-12 (DMEM/F12; Sigma-Aldrich) (17.5 mM glucose) supplemented with 2 g/l BSA (Sigma-Aldrich), 10 ng/ml keratin growth factor (Sigma-Aldrich), 10 mM nicotinamide, 2.5 mM L-glutamine, ITS (5 mg/ml Insulin, 5 mg/ml human transferrin and 5 μg/l Selenite; BD Biosciences, Franklin Lakes, NJ, USA), 100 U/ml penicillin and 100 μg/ml streptomycin (Laboratorios Normon). After 3–4 days in culture, cells clustered into three-dimensional structures, termed ductal pancreatospheres (DPS).

### Animals

Male athymic nude-Foxn1^nu^ mice (Envigo, Horst, The Netherlands) housed under specific pathogen-free conditions on a 12-h light cycle with free access to food and water were used as recipients of human islet transplantation. Mice were maintained in the animal facility of the Bellvitge Biomedical Research Institute (IDIBELL) following to the guidelines and recommendations of the Association for Assessment and Accreditation of Laboratory Animal Care International (AAALAC International, accreditation number 1155). Mice were sacrificed by pentobarbital overdose. Animal experimental procedures were reviewed and approved by the Ethical Committee for Animal Experimentation of the IDIBELL (identification number DAAM 4588).

### Experimental design

#### Islet and ductal cell co-culture

**Fig 1:**
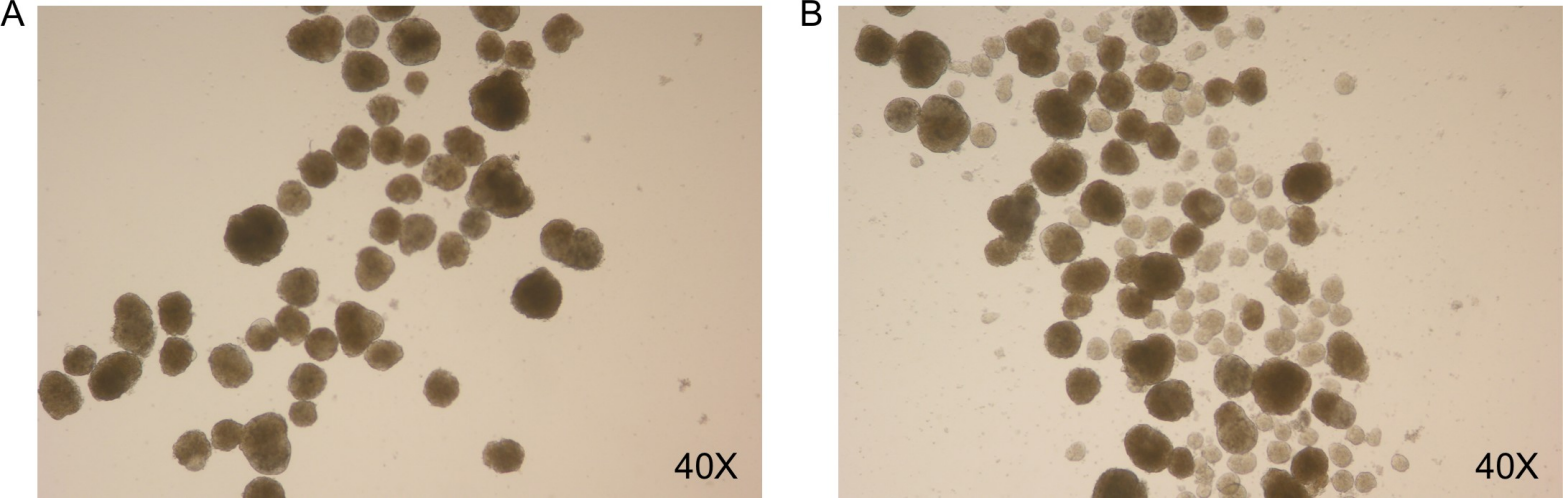
Islets and islets + DPS preparations. Representative image of 100 islets cultured alone (A) or with 200 DPS (B). Magnification 40X.

Preparations with 100 handpicked islets, 300 DPS, and islets + DPS (100 islets + 75 DPS, or 100 islets + 200 DPS) (Fig 1) were cultured in CMRL-1066 medium supplemented with 10% ABO compatible human serum. The characteristics of the preparations were determined using images of islets and islets + DPS preparations taken on day 0 with an inverted microscope (Nikon Eclipse TS 100, Tokyo, Japan). The diameter of islets and DPS (at least 60 islets and 100 DPS per isolation) was measured with ImageJ software (National Institutes of Health, Bethesda, MD, USA). After 48h in culture, β-cell function (glucose-stimulated insulin secretion), β-cell apoptosis, and gene expression were determined, and supernatants were collected and stored at -80°C. In supernatants, IL-1β was quantified by ELISA (Human IL-1β High Sensitivity ELISA, eBioscience, San Diego, CA, USA). For immunohistochemical analysis, preparations were pelleted, fixed overnight in 4% paraformaldehyde-PBS (PFA; Merck KgaA, Darmstadt, Germany), and processed for paraffin embedding. For gene expression analysis, the preparations were processed for RNA extraction.

#### Islet and ductal cell co-transplantation

Eight hundred handpicked islets (islets group n = 14) or 800 handpicked islets and 600 DPS (islets + DPS group, n = 14) were transplanted under the kidney capsule of nude-Foxn1^nu^ mice. Briefly, islets and islets + DPS preparations were placed into a polyethylene tubing PE-50 (Becton Dickinson, Sparks, MD, USA) with the help of a 1 ml Hamilton syringe (Hamilton, Reno, NE, USA). The tubing was folded and centrifuged until 700 g and stopped without brake to pellet the preparations. Mice were anesthetized by isoflurane inhaled and the left kidney was exposed through lumbar incision. A capsulotomy was performed in the lower pole of the kidney and the islets or islets + DPS were carefully injected. The capsulotomy was cauterized with a disposable low-temperature cautery pen (AB Medica, S.A., Barcelona, Spain) and the lumbar incision was sutured. Analgesia (meloxican) was administrated during the surgical procedure, and every 24 hours for three days after transplantation. On day 3 after transplantation, 10 grafts (5 in each group) were harvested and processed for RNA extraction. Ten to 14 days after transplantation, the remaining transplanted mice (n = 18) were injected intraperitoneally with five consecutive daily-doses of 70 mg/kg body weight streptozotocin (STZ; Sigma-Aldrich) as described in recently reported new model for STZ-diabetes induction after human islet transplantation [28]. A control group of non-transplanted mice was also injected with STZ (n = 4). Blood glucose was determined between 9:00 and 11:00 A.M. in non-fasting conditions. Animals were considered hyperglycemic when blood glucose values were >11.1 mM on two consecutive measurements. Thirty days after the last STZ injection, the grafts were harvested, immediately immersed in 4% PFA, fixed overnight, weighed after removal of any excess PFA by capillary action, and processed for paraffin embedding.

### Glucose-stimulated insulin secretion

Groups of 10 islets handpicked from islets and from islets + DPS preparations were pre-incubated in 1 ml fresh KRBH buffer containing 2.8 mM glucose for 1 h at 37°C, and sequentially incubated with 1 ml of KRBH buffer containing 2.8 and 20 mM glucose for an additional hour with continuous shaking. Supernatants were collected and stored at –80°C until assayed for insulin content (Mercodia AB, Uppsala, Sweden). The simulation index was calculated as the secreted insulin ratio between stimulated (20 mM) and basal (2.8 mM) glucose concentrations.

### Insulin and DNA content

After the insulin secretion assay, islets were sonicated. For insulin content, the homogenate was extracted with acid-ethanol solution. For DNA quantification, an aliquot of the homogenate was collected and stored at −80°C. DNA was determined by fluorimetry using 3.33 μg/ml Hoechst 33258 (Sigma-Aldrich) (excitation wave length 356 nm, and emission wave length 448 nm) on a fluorescence spectrophotometer (FLUOstar Omega, BMG Labtech, Ortenberg, Germany).

### Immunostaining

#### β-cell apoptosis

Sections from cultured islets and islets + DPS preparations were double stained by immunofluorescence for apoptotic nuclei with the terminal deoxynucleotidyl transferase biotin-dUTP nick-end labeling (TUNEL) technique (ApopTag Plus Fluorescein *In Situ* Apoptosis Detection Kit; Merk Millipore), and for insulin using a rabbit anti-human insulin antibody (Santa Cruz Biotechnology Inc., Dallas, TX, USA) (final dilution 1:100). Preparations were visualized by immunofluorescence with donkey anti-rabbit IgG conjugated with Alexa Fluor 555 (Life Technologies) (final dilution 1:400). Nuclei were stained with 300 nM DAPI (Life Technologies). β-cell apoptosis was expressed as percentage of TUNEL-positive β-cells with pyknotic nucleus. A minimum of 1,200 cells per sample was counted.

#### Morphometry

Sections from islets and islets + DPS preparations, and from grafts were double stained for insulin using the rabbit anti-human insulin antibody (final dilution 1:100) and for cytokeratin 19 (CK19) using a mouse anti-human CK19 antibody (DakoCytomation, Carpinteria, CA, USA) (final dilution 1:80). Consecutive sections were stained using a cocktail of antibodies including rabbit anti-human glucagon (Cell Signaling Technology, Danvers, MA, USA) (final dilution 1:100), rabbit anti-human somatostatin (DakoCytomation) (final dilution 1:500) and rabbit anti-human pancreatic polypeptide (Merk Millipore) (final dilution 1:2000). Islets and islets + DPS preparations were also stained using a rabbit anti-amylase antibody (Sigma-Aldrich) (final dilution 1:50). Preparations were visualized by immunofluorescence with donkey anti-rabbit IgG conjugated with Alexa Fluor 555 or 488 (Life Technologies) (final dilution 1:400) and goat anti-mouse IgG conjugated with Alexa Fluor 555 (Life Technologies) (final dilution 1:400). Nuclei were stained with 300 nM DAPI. Relative cell area was determined by point-counting morphometry on immunostained sections of islets, islets + DPS and grafts using a point grid to obtain the number of intercepts over β-cells, endocrine non-β-cells, acinar cells, ductal cells, and over other tissue. The relative area of each cell type was calculated by dividing the intercepts over each specific cell type by intercepts over total tissue [29,30]. To validate the relative area as volume, we used a nomogram relating number of points counted to volume density and expected relative standard error in percentage of mean (<10%) and established the number of intercepts needed for a representative sampling [30]. In grafts, β-, endocrine non-β-, and ductal cell mass, was obtained by multiplying the respective relative volume by the graft weight. All measurements were performed by a blinded observer.

### Quantitative PCR

RNA was isolated from culture preparations and from grafts harvested on day 3 after transplantation. Total RNA was extracted with RNeasy Mini Kit (Qiagen, Crawley, UK) according to the manufacturer’s instructions. RNA quality was assessed with the Bioanalyzer 2100 (Agilent Technologies, Inc., Palo Alto, CA, USA). The RNA Integrity Number score ranged from 6.5 to 9.9. cDNA synthesis was performed from 2 μg of total RNA using High Capacity cDNA Reverse Transcription Kit (Applied Biosystems, Foster, CA, USA).

PCR was run in a 7900HT fast Real-Time PCR system (Applied Biosystems) with 384-well optical plates. Reactions were performed using TaqMan Gene Expression Assays and TaqMan Gene Expression Master Mix (Applied Biosystems) following the manufacturer protocol in a final volume of 20 μl with 20 ng of cDNA in each reaction. A full listing of assays (Applied Biosystems), gene names, and assay identification number is given in S1 Table. Relative quantities (RQ) were calculated using the software Gene Expression Suite v1.0.3 (Applied Biosystems) and the 2^-ΔΔCT^ analysis method with 60S acidic ribosomal protein P0 (*RPLP0*) as the endogenous control. RQs were normalized to give a mean of 1 for islets at day 0 and for grafts harvested from islets group.

### Statistical analyses

Results are expressed as means ± SEM. Statistics were performed using GraphPad Prism 5 software (GraphPad Software Inc, La Jolla, CA, USA). Differences among data normally distributed were evaluated using the Student’s *t-*test or the one-way analysis of variance (ANOVA) and Tukey’s test for *post hoc* analysis. Data not normally distributed was analyzed using the Wilcoxon matched paired test, Mann-Whitney test or the Kruskal-Wallis one-way ANOVA and *post hoc* Dunn’s test. A *p* value of <0.05 was considered significant.

## Results

### Islet and ductal cell preparation characteristics

The islet-enriched fractions collected after islet isolation were cultured for 4–6 days and the islets were handpicked to further increase the purity of the preparations. Based on morphometric analysis of immunostained preparations, the endocrine and ductal cell relative areas of these purified islet preparations were 77.8 ± 4.9% and 6.5 ± 1.5% respectively (Table 1). The mean islet diameter was 164 ± 6.0 μm. The low gene expression of the ductal cell marker CA*II* was in agreement with the low ductal cell content of the preparations (S1A and S1D Fig).

**Table 1:** Endocrine and exocrine cell relative area in islets and islets + DPS preparations.

	β-cells	Endocrine non-β-cells	Ductal cells	Acinar cells	Other cell types
**Islets**	48.0 ± 1.8%	29.7 ± 3.1%	6.50 ± 1.5%	9.87 ± 3.7%	5.83%
**Islets** **+ DPS**	46.4 ± 4.3%	25.6 ± 4.4%	13.8 ± 2.0%	10.1 ± 3.3%	4.13%

Eight hundred handpicked islets (islets) and 800 handpicked islets and 600 DPS (islets + DPS) collected as for transplantation were pelleted, fixed, and embedded in paraffin. Sections were immunostained and the relative area of β-, endocrine non-β-, ductal and acinar cells was determined by point-counting morphometry. Values are means ± SEM, (n = 3).

The exocrine, islet-depleted, fractions collected after islet isolation were dissociated into single cells, and ductal cells were purified by MACS. After MACS, the percentage of ductal cells increased from 45.8 ± 3.6% to 78.7 ± 8.4%, and the percentage of β-cells was reduced from 0.5 ± 0.3% to 0.2 ± 0.1%. After three days in culture, cells clustered into three-dimensional structures of ductal pancreatospheres (DPS) with a mean diameter of 84.9 ± 5.1 μm and largely composed of CK19+ ductal cells (88.4 ± 2.2%) (S1B Fig). Insulin gene expression was almost undetectable in DPS preparations (S1C Fig). The endocrine and ductal cell relative area of the islets + DPS preparations used in transplantation experiments were 72.0 ± 8.7% and 13.8 ± 2.0% respectively (Table 1). The acinar cell relative area was similar in islets and in islets + DPS preparations (Table 1).

### Effects of ductal cells on cultured islets

#### β-cell function

After 48h in culture, insulin content was similar in 10 islets handpicked from the islets cultured alone (241 ± 18.2 ng insulin/μg DNA), and from islets cultured with DPS (232 ± 19.8 ng insulin/μg DNA) preparations (n = 6). Islets co-cultured with DPS showed a lower insulin stimulation index than islets cultured alone (islets + DPS: 4.02 ± 0.46; islets: 5.59 ± 0.93; *p* = 0.048; n = 6). The lower insulin stimulation index of islets cultured with DPS was due to the higher baseline insulin secretion at 2.8 mM glucose (islets + DPS: 8.72 ± 2.07 ng insulin/μg DNA·h; islets 5.59 ± 1.05 ng insulin/μg DNA·h; n = 6). A subanalysis of islets cultured with DPS showed that the insulin stimulation index tended to be lower with the higher number of DPS, although differences did not reach statistical significance (100 islets + 75 DPS: 4.31 ± 0.55; 100 islets + 200 DPS: 3.72 ± 0.47; *p* = 0.25; n = 6).

#### β-cell apoptosis

Ductal cells had no detectable effect on β-cell apoptosis. After 48h in culture, β-cell apoptosis was similar in islets cultured alone and islets cultured with DPS (islets: 0.74 ± 0.31%; 100 islets + 75 DPS: 0.68 ± 0.31%; 100 islets + 200 DPS: 0.68 ± 0.38%; n = 5, all groups).

#### Gene expression

**Fig 2:**
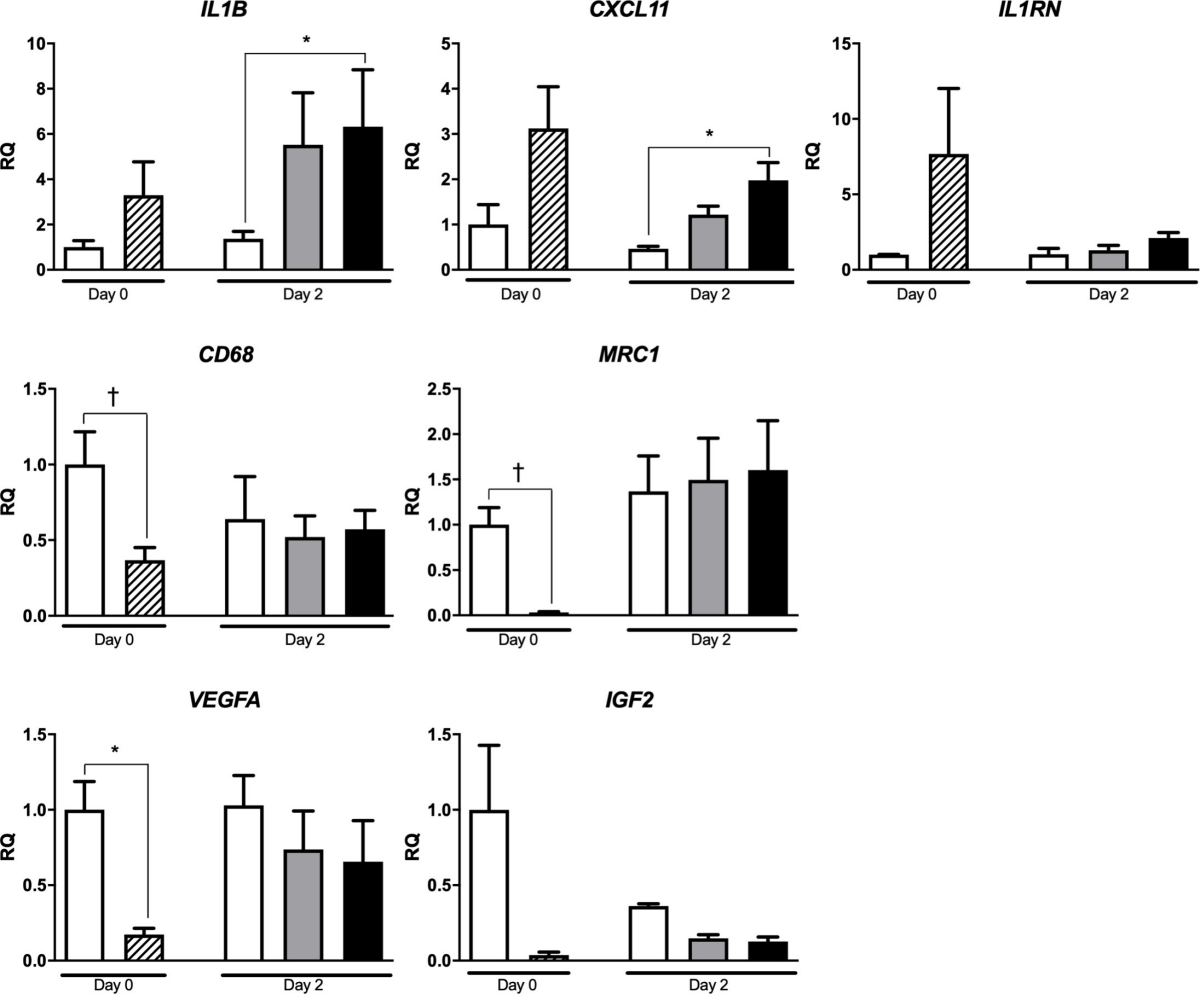
Gene expression in cultured islets, ductal cells and islets + DPS preparations. Islets (white bar), ductal cells (hatched bar), 100 islets + 75 DPS (grey bar), and 100 islets + 200 DPS (black bar). Values are means ± SEM, (*IL1B* and *IL1RN* n = 5; *CXCL11* n = 4; *CD68*, *MRC1*, *VEGFA* and *IGF2* n = 4 on day 0, and n = 3 on day 2). † *p*<0.05, * *p*≤0.01. Wilcoxon matched paired test (day 0), and Kruskal Wallis one-way ANOVA and *post hoc* Dunn’s test (day 2) for *IL1B*, *IL1RN*, *CXCL11* and *IGF2* analysis. Paired Student’s *t*-test (day 0), and one-way ANOVA and *post hoc* Tukey’s test (day 2) for *CD68*, *MRC1* and *VEGFA* analysis. RQ, relative quantity.

Baseline expression of *IL1B*, *CXCL11* and *IL1RN* genes was higher in DPS than in islets preparations, although the difference did not reach statistical significance (Fig 2). After 48h in culture, the expression of *IL1B* and *CXCL11* genes, was higher in islets + DPS preparations than in islets. Baseline expression of macrophage markers *CD68* and *MRC1* and angiogenic factor *VEGFA* was lower in DPS than in islets. The lower *IGF2* expression in DPS was not statistically significant. After 48h in culture, the expression of *CD68*, *MRC1*, *VEGFA* and *IGF2* was similar in islets and islets + DPS groups.

#### IL-1β secretion

After 48h in culture, IL-1β was below the detection limit (0.05 pg/ml) in all islet alone preparations and was detected in 12.9%, 17.9% and 37.5% of supernatants collected from 100 islets + 75 DPS, 100 islets + 200 DPS and DPS preparations respectively (n = 31 for islets and 100 islets + 200 DPS, n = 28 for 100 islets + 75 DPS, n = 8 for DPS).

### Effects of ductal cells on islet transplantation

#### Blood glucose

**Fig 3:**
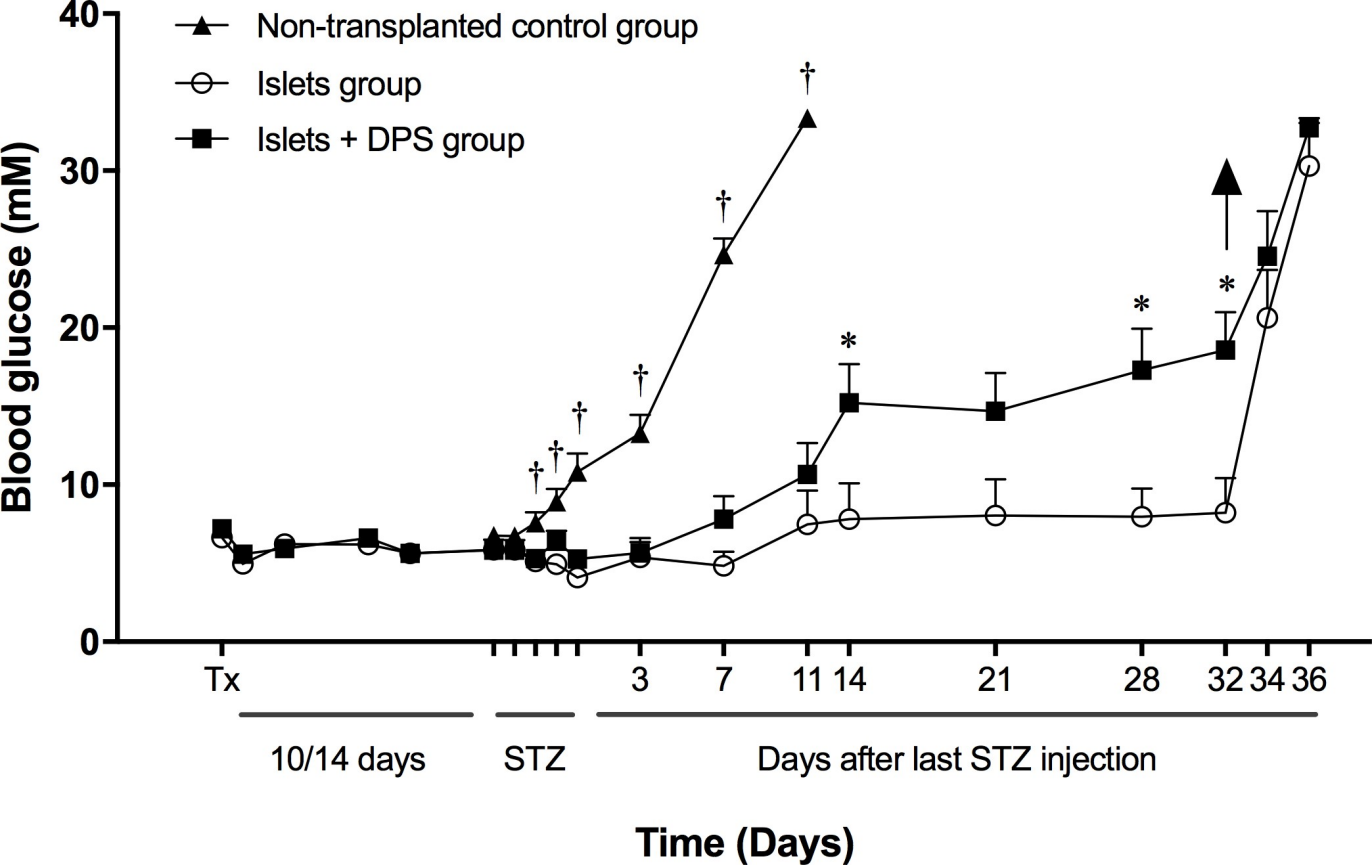
Blood glucose levels of mice transplanted with islets and islets + DPS, and treated with STZ. Mice were transplanted with 800 human islets (n = 9), with 800 islets + 600 DPS (n = 9), or were non-transplanted (n = 4). At the end of the follow-up, 3 mice transplanted with human islets alone and 6 mice co-transplanted with islets and ductal cells had developed hyperglycemia. *Tx* indicates transplantation day; *STZ*: streptozotocin administration; arrow: graft removal. Values are means ± SEM. † *p*<0.05 *vs* all other groups (ANOVA and *post hoc* Tukey’s test on STZ injection days, and days 3, 7 and 11 after last STZ injection); *p<0.05 *vs* islets group (unpaired Student’s *t*-test, from day 14 after last STZ injection to the end of study).

After STZ injection, normoglycemia was initially maintained in all transplanted animals, whereas all non-transplanted animals developed hyperglycemia. On day 30 after STZ injection (6 weeks after transplantation), 33% of animals transplanted with human islets alone and 67% of those co-transplanted with islets and ductal cells had developed hyperglycemia (Fig 3). All transplanted and STZ-injected animals that remained normoglycemic at the end of follow-up developed hyperglycemia when the graft was harvested. Hyperglycemia was detected earlier in co-transplanted than in islet-alone transplanted animals (11.8 ± 1.37 days *vs* 15.3 ± 2.96 days after the last STZ injection). Thus, the presence of ductal cells in islet preparations was associated with an increased and accelerated failure of the graft.

#### Graft morphometry

**Fig 4:**
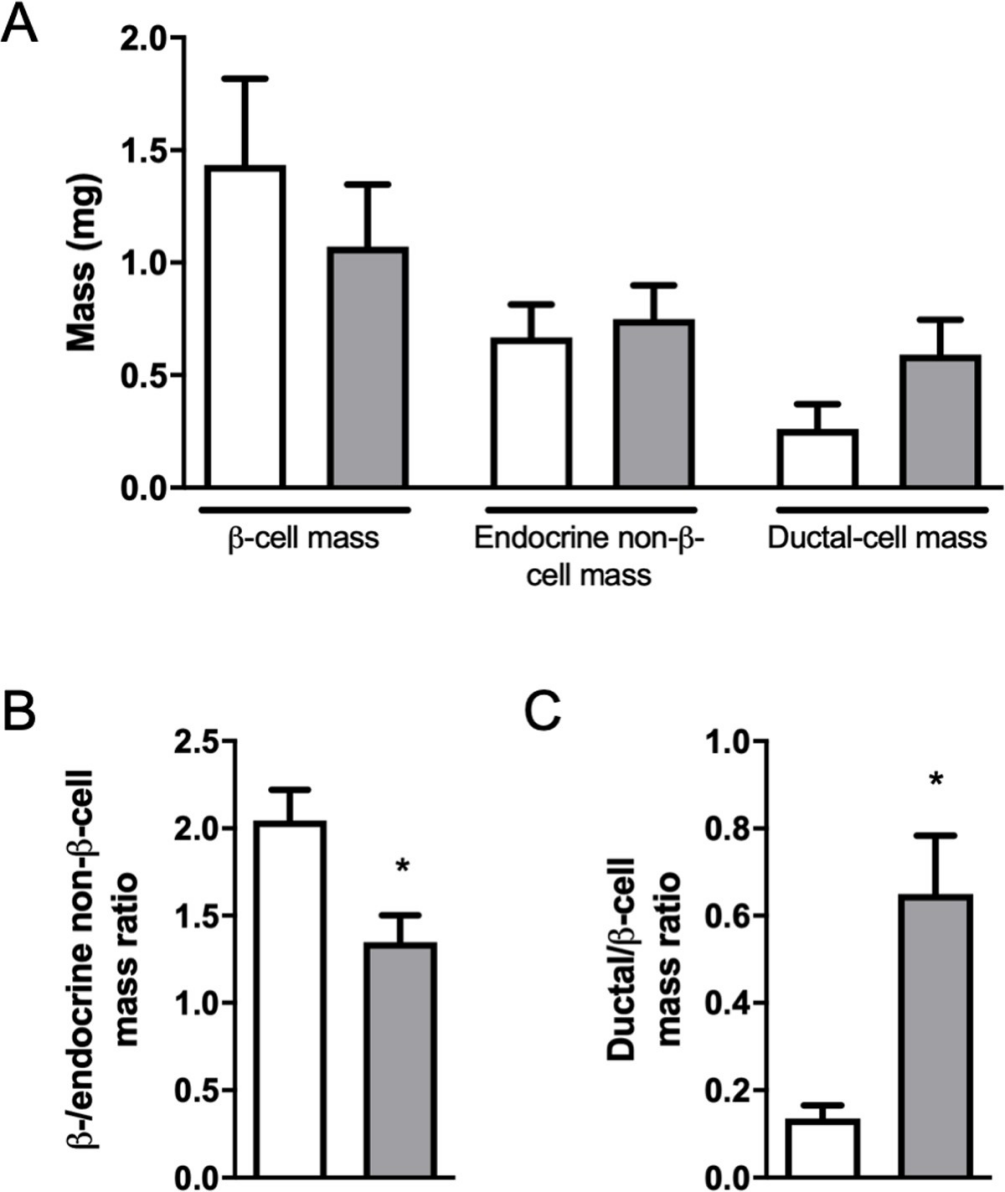
Graft endocrine and ductal cell mass. β-cell, endocrine non-β-cell and ductal-cell mass in grafts harvested 6 weeks after transplantation (A). Ratio between β-cell and endocrine non-β-cell mass (B). Ratio between ductal cell and β-cell mass (C). Islet group (white bars); islets + DPS group (grey bars). Values are mean ± SEM, (n = 9). * *p*<0.001 (Mann-Whitney test).

β-cell mass was 26% lower in grafts from islets + DPS group (1.07 ± 0.28 mg) than in grafts from islets group (1.44 ± 0.38 mg) at the end of follow-up, although the difference was not statistically significant (*p* = 0.297) (Fig 4A). The endocrine non-β-cell mass was similar in both groups (islets: 0.75 ± 0.15 mg; islets + DPS 0.67 ± 0.15 mg). The ratio β-/endocrine non-β-cell mass was significantly reduced in grafts of mice co-transplanted with islets and ductal cells (islets + DPS: 1.35 ± 0.15; islets: 2.05 ± 0.18; *p* = 0.006) (Fig 4B). In co-transplanted grafts, the ductal cell mass was 2.3 times higher than in islets grafts (islets + DPS: 0.59 ± 0.16 mg; islets: 0.26 ± 0.11 mg; *p* = 0.062) and the ratio ductal/β-cell mass was 5 times higher (islets + DPS: 0.65 ± 0.13; islets: 0.14 ± 0.03, *p* = 0.001) (Fig 4A and 4C).

#### Gene expression

**Fig 5:**
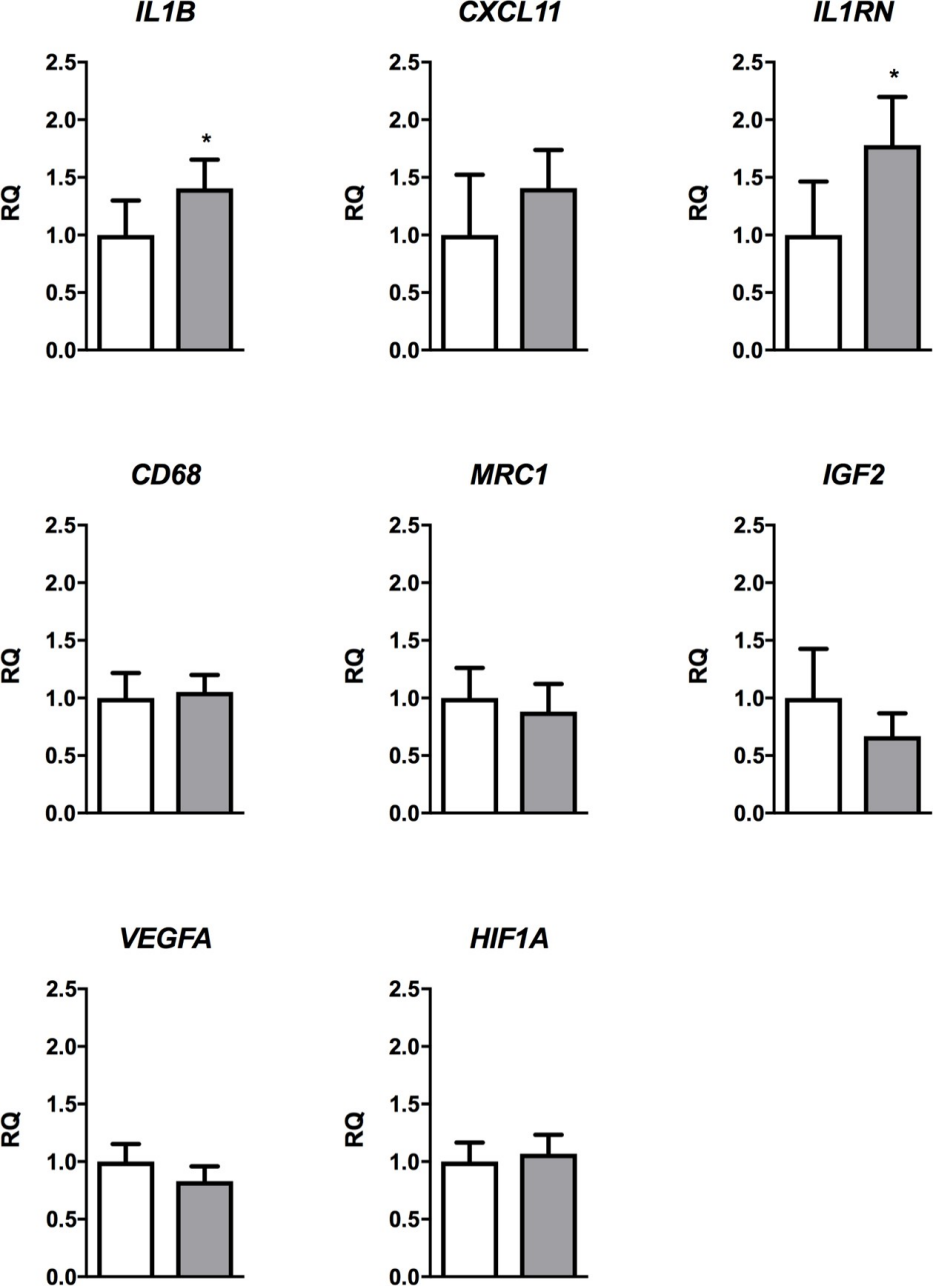
Gene expression in grafts. Islets (white bar) and islets + DPS (grey bar) grafts harvested on day 3 after transplantation. Values are mean ± SEM, (n = 5). * *p*<0.01 (paired Student’s *t*-test). RQ, relative quantity.

The gene expression pattern in islets and islets + DPS grafts was similar to that found in culture experiments. *IL1B* and *IL1RN* expression was significantly higher in grafts of islets + DPS group (Fig 5). As in culture experiments, gene expression of macrophage markers *CD68* and *MRC1*, *VEGFA* and *IGF2* was similar in grafts from islets and islets + DPS groups. The expression of hypoxia gene *HIF1A* was also similar.

## Discussion

In this study, we have found that islet preparations had a higher insulin stimulation index *in vitro* and achieved a better metabolic outcome after transplantation than islet preparation enriched in ductal cells. Islets co-cultured with ductal cells, and grafts transplanted with islets and ductal cells showed a higher expression of inflammatory molecules than islet preparations or islet grafts suggesting that inflammation could mediate the deleterious effects of ductal cells on islets.

We found a higher baseline insulin secretion, an indicator of poor islet quality [31], and a lower insulin stimulation index in response to glucose in islets cultured with ductal cells. The reduction in insulin stimulation index in islets + DPS preparations showed a tendency towards a more profound impairment in preparations with higher number of DPS, suggesting that the effect of ductal cells on islets is dose-dependent. The effect of pancreatic ductal cells contaminating islet preparations on β-cell function has been scarcely investigated. In a recent study, Henquin observed a high baseline insulin secretion, a blunted first phase response, and a lower stimulation index in human islet preparations with a lower purity [23]. Purity of islet preparations was also shown to influence baseline insulin secretion in a large sample of human islet preparations [32]. However, these studies did not analyze whether the negative effect was due to the presence of acinar, ductal or other cell types contaminating the islet preparation. In our study, the percentage of acinar cells was similar in islets and islets + DPS preparations, therefore excluding a significant contribution of acinar cell contamination to the impaired islet function of islets + DPS preparations. Although some studies have reported a neutral, or even positive, effect of ductal cells on islet insulin secretion *in vitro*, the interpretation of these results is limited by the lack of correction of insulin secretion and the absence of an insulin response to glucose stimulation in control islets [33,34].

Streptozotocin-injected animals transplanted with purified human islet preparations showed a better metabolic evolution than those transplanted with a combination of islets and ductal cells. In a recent study, co-transplantation of ductal cells and human islets to diabetic mice did not significantly modify the metabolic outcome, although the achievement of normoglycemia was delayed in co-transplanted group [16]. In clinical islet transplantation, the effect of ductal cells has only been analysed in a few retrospective, non-controlled, studies. Street et al. identified a positive correlation between the number of transplanted ductal cells and insulin secretion two years after transplantation [12]. A recent study reported a better long-term outcome with the transplantation of less pure (mean purity 45%) than more pure (mean purity 60%) islet preparations [24], and the authors suggested that the presence of more ductal cells in the less pure preparations could have been beneficial. However, ductal cell mass in the preparations was not quantified, and other studies have reported a negative impact of exocrine volume in human islet transplantation [35].

The negative effect of ductal cells on human islets *in vitro* and after transplantation may be a consequence of increased inflammation. After 48 hours in culture, the expression of cytokine *IL1B* and chemokine *CXCL11* genes was higher in islets + DPS than in islets alone preparations. Concordantly, IL-1β was detectable in the supernatant of DPS and of islets + DPS preparations, but not in islet alone preparations. IL-1β has well known deleterious effects on β-cell function [36–38], and IL-1β secreted by ductal cells may have contributed to impair insulin secretion in islets co-cultured with DPS. Similar to the *in vitro* results, *IL1B* and *IL1RN* gene expression was significantly higher in islets + DPS grafts than in islets grafts. IL-1β contribution to graft dysfunction has been reported in xenogeneic islet transplantation in non-obese diabetic mice [39] and in syngeneic islet transplantation [40]. In previous studies, we identified the presence of non-specific inflammation in syngeneic islet grafts with the contribution of several cytokines, among them IL-1β [41]. We also showed that IL-1 receptor antagonist protein overexpression in transplanted islets improved the outcome of the transplantation, confirming the deleterious effect of IL-1β on islet grafts [42]. Thus, IL-1β secreted by ductal cells may contribute to the poorer outcome of islets co-transplanted with ductal cells. We identified the increased graft inflammation using an immunodeficient recipient for transplantation, and we speculate that in an immunocompetent recipient the inflammatory molecules secreted by ductal cells could activate or recruit immune cells infiltrating the graft and further enhance the inflammation.

The expression of macrophage markers (*CD68* and *MRC1*) was lower in ductal cells than in islets, and it was similar in islets and islets + DPS grafts, supporting the notion that macrophage infiltration did not play a role in the increased inflammation observed in co-transplanted grafts. The low expression of *MRC1* in ductal cells, a marker of the alternative activated M2 macrophages, involved in maintenance of homeostasis and tissue remodeling [43], suggests that the capacity of ductal cells to contribute to tissue repair in islet transplantation may be limited.

In addition to increased inflammation, other factors could have contributed to the poor outcome of co-transplanted grafts. The transplantation, in close contact, of islets and ductal cells under the kidney capsule could lead to a competition for local nutrient factors or oxygen and increase the hypoxia of islets. However, the similar expression of the hypoxia gene *HIF1A* in islets and islets + DPS grafts does not support the presence of an increased hypoxia in co-transplanted grafts.

A beneficial effect of ductal cells could have been expected based on previous reports indicating that they secrete the angiogenic factors VEGF and IL-8 [19,20] and the growth factor IGF-2 [22]. However, Xiao et al. reported that the levels of *VEGF* transcript in ductal cells were 42% lower than in β-cells [20]. We have also found that *VEGFA* expression is lower in human ductal cells than in islets. Ilieva et al. reported a reduced central necrosis and apoptosis in islets co-cultured with ductal cells that they suggested could be mediated by IGF-2 secreted by ductal cells [22]. However, and similar to our results, Ichii et al. [16] found no differences in β-cell viability in human islets cultured with or without ductal cells. We have found a very low expression of *IGF2* in human ductal cells compared to islets, suggesting that human ductal cells are not a relevant source of IGF-2. Finally, β-cell neogenesis from ductal cells [21] could increase the graft β-cell mass. Although, we did not specifically address this possibility, the lower β-cell mass in co-transplanted grafts at the end of follow-up does not support a relevant role of human ductal cells as a source of new β-cells in the initial weeks after transplantation.

Our study has several strengths, in particular, the additional purification and careful quantification of islets and ductal cells in the preparations that allowed the transplantation of a pre-established well-defined islet/ductal cell ratio. The importance of strict characterization of islet preparations has been recently emphasized [44]. The purity of the islet preparations was enhanced by the additional islet handpicking performed in the islet-enriched fractions collected after the COBE 2991 purification. The ductal cell preparations were purified by magnetic cell sorting of the exocrine fractions. The large number of transplants and the parallel transplantation of islets and islets + DPS preparations isolated from the same donor are additional strengths of the study. Finally, to the best of our knowledge, this is the first study in which the cellular composition and gene expression in grafts co-transplanted with islets and ductal cells has been determined. Our study has also limitations. The DPS may not have the same characteristics of native ductal cells, even though we confirmed that DPS stained positive for CK19 and expressed *CAII*, both well stablished ductal cell markers. In addition, the gene expression analysis was restricted to a fraction of genes expressed in pancreatic ductal cells that could have an effect on islet cells, and we can not exclude a significant contribution of other genes to the deleterious effect of ductal cells on β-cells.

In summary, we have found that increasing the percentage of ductal cells in islet preparations has a deleterious effect on islet transplantation outcome, probably by increasing local inflammation at the grafted site. The results do not support the enrichment of islet preparations with ductal cells for transplantation purposes.

## Supporting information

S1 Fig Islets and ductal cells preparation characteristics. Representative image of insulin (green) and CK19 (red) double immunofluorescence of handpicked islet preparations (A) and of DPS (B). Gene expression of *INS* (C) and *CAII* (D) in islets (white bar) and in DPS (black bar) at baseline. Values are means ± SEM, n = 9. RQ, relative quantity. Scale bar = 100 μm.
(TIFF)

S1 Table Gene expression assays used for real-time qPCR. (DOC)
