# Supplementary figures and images for: Pancreatic ductal cells may have a negative effect on human islet transplantation
Source: PLoS One. 2019 Jul 19;14(7):e0220064. doi: 10.1371/journal.pone.0220064 (PMC6641198; doi:10.1371/journal.pone.0220064)

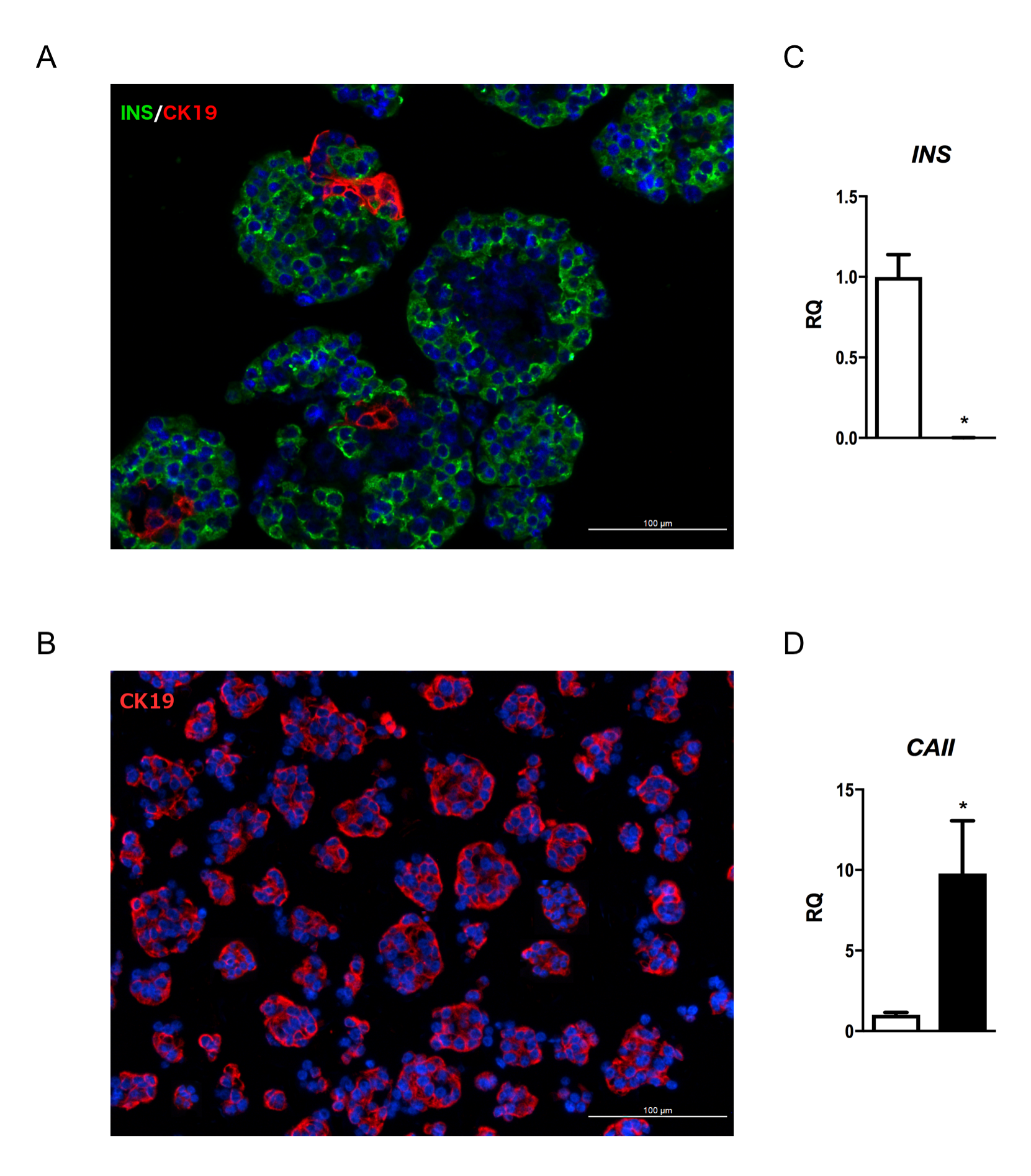

Supplement: S1 Fig — Representative image of insulin (green) and CK19 (red) double immunofluorescence of handpicked islet preparations (A) and of DPS (B). Gene expression of INS (C) and CAII (D) in islets (white bar) and in DPS (black bar) at baseline. Values are means ± SEM, n = 9. RQ, relative quantity. Scale bar = 100 μm. (TIFF) [file pone.0220064.s001.tiff]
