# Supplementary material for: Pancreatic ductal cells may have a negative effect on human islet transplantation
Source: PLoS One. 2019 Jul 19;14(7):e0220064. doi: 10.1371/journal.pone.0220064 (PMC6641198; doi:10.1371/journal.pone.0220064)
Supplement: S1 Table — (DOC) [file pone.0220064.s002.doc]

**S1 Table. Gene expression assays used for real-time qPCR**.

| **Gene name** | **Gene symbol** | **Assay ID** |
| --- | --- | --- |
| **Carbonic anhydrase II** | CAII | Hs01070108_m1 |
| **CD68 molecule** | CD68 | Hs02836816_g1 |
| **Chemokine (C-X-C motif) ligand 11** | CXCL11 | Hs04187682_g1 |
| **Hypoxia inducible factor 1, alpha subunit (basic helix-loop-helix transcription factor)** | HIF1A | Hs00153153_m1 |
| **Insulin** | INS | Hs00355773_m1 |
| **Insulin-like growth factor 2 (somatomedin A)** | IGF2 | Hs04188276_m1 |
| **Interleukin 1 receptor antagonist** | IL1RN | Hs00893626_m1 |
| **Interleukin 1, beta** | IL1B | Hs00174097_m1 |
| **Mannose receptor, C type 1 (CD206)** | MRC1 | Hs00267207_m1 |
| **Ribosomal protein, large, P0** | RPLP0 | Hs99999902_m1 |
| **Vascular endothelial growth factor A** | VEGFA | Hs00900055_m1 |
